# fMRI Brain-Computer Interface: A Tool for Neuroscientific Research and Treatment

**DOI:** 10.1155/2007/25487

**Published:** 2007-11-25

**Authors:** Ranganatha Sitaram, Andrea Caria, Ralf Veit, Tilman Gaber, Giuseppina Rota, Andrea Kuebler, Niels Birbaumer

**Affiliations:** ^1^Institute of Medical Psychology and Behavioral Neurobiology, Eberhard-Karls-University of Tübingen, 72074 Tübingen,Germany; ^2^Max Planck Institute for Biological Cybernetics, P.O. Box 21 69, 72076 Tübingen, Germany; ^3^Institute for Natural Language Processing, University of Stuttgart, 70174 Stuttgart, Germany; ^4^National Institute of Health (NIH), NINDS, Human Cortical Physiology, Bethesda, MD 20892-1428, USA

## Abstract

Brain-computer interfaces based on functional magnetic resonance imaging (fMRI-BCI) allow volitional control of anatomically specific regions of the brain. Technological advancement in higher field MRI scanners, fast data acquisition sequences, preprocessing algorithms, and robust statistical analysis are anticipated to make fMRI-BCI more widely available and applicable. This noninvasive technique could potentially complement the traditional neuroscientific experimental methods by varying the activity of the neural substrates of a region of interest as an independent variable to study its effects on behavior. If the neurobiological basis of a disorder (e.g., chronic pain, motor diseases, psychopathy, social phobia, depression) is known in terms of abnormal activity in certain regions of the brain, fMRI-BCI can be targeted to modify activity in those regions with high specificity for treatment. In this paper, we review recent results of the application of fMRI-BCI to neuroscientific research and psychophysiological treatment.

## 1. INTRODUCTION

Brain-computer interfaces (BCIs) enable control of computers and of external devices with regulation of brain activity alone (Birbaumer et al. [1], Donoghue [2], Wolpaw et al. [3], Nicolelis [4], Wolpaw and McFarland [5], Hochberg and Donoghue [6]). Two different traditions of BCI research have dominated the field: invasive BCI, based on animal studies and realized with implanted electrodes, and noninvasive BCI, primarily using electroencephalography (EEG). Invasive multielectrode BCIs in animals enabled execution of reaching, grasping, and force control from spike patterns and extracellular field potentials. Clinical applications have been derived predominantly from noninvasive approaches: communication for the completely paralyzed and locked-in patients using slow cortical potentials, sensorimotor rhythm, and the P300 event-related potential, and restoration of movement and cortical reorganization in high spinal cord lesions and chronic stroke.

EEG-BCIs have certain drawbacks. Mainly, EEG provides only a low spatial resolution and ambiguous localization of neuronal activity, since underlying electric sources need to be reconstructed from the distribution of electric potentials across the scalp (Weiskopf et al. [7]). A BCI based on real-time fMRI allows for noninvasive recording of neuronal activity across the entire brain with relatively high spatial resolution and moderate temporal resolution (in the range of millimeters and seconds, resp.). Unlike EEG-BCI, fMRI-BCI allows brain activity in very specific parts of cortical and subcortical regions of the brain, for example, the left anterior insula, to be extracted and used for online feedback (Caria et al. [8]). However, major disadvantages of fMRI-BCI are its high cost and complexity of development and usage. With the wide-spread use of MRI systems in the clinics and research centres, and the emergence of real-time fMRI data processing and analysis tools such as turbo-brain voyager (Brain Innovations, Maastricht, The Netherlands) and TurboFIRE (Sefan Posse, NM, USA), fMRI-BCI might become more accessible in the future.

Despite the fact that BOLD is an indirect measure, there is growing evidence for a strong correlation between the BOLD signal and electrical brain activity. Studies have characterized the relationship between localized increases in neuronal activity and the corresponding increase in BOLD (Logothetis et al. [9], Shmuel et al. [10]), making it possible to interpret positive functional responses in terms of neural changes. These results constitute a convincing basis for using fMRI in BCI studies. With innovations in high-performance magnetic resonance scanners and computers, and developments in techniques for faster acquisition, processing and analysis of MR images, real-time fMRI has recently become a possibility. With improvements in real-time fMRI, a novel type of noninvasive fMRI-BCI has emerged.

Studies that have been reported so far (Yoo and Jolesz [11], Posse et al. [12], Weiskopf et al. [13], DeCharms et al. [14], Weiskopf et al. [15], Yoo et al. [16], DeCharms et al. [17], Sitaram et al. [18], Caria et al. [19], Rota et al. [20], Veit et al. [21]) have demonstrated that human subjects using real-time fMRI can learn voluntary self-regulation of localized brain regions. These studies manipulated different cortical and subcortical areas, namely, supplementary motor area (SMA) (Wagner and Barrett [22], Weiskopf et al. [15], Sitaram et al. [18]), sensorimotor area (Yoo and Jolesz [11], DeCharms et al. [14], Yoo et al. [16]), posterior part of the superior temporal gyrus (Yoo et al. [16]), medial superior frontal gyrus (Yoo et al. [16]), parahippocampal place area (PPA) (Weiskopf et al. [15]), the anterior cingulate cortex (ACC) (Weiskopf et al. [13], temporal gyrus (Yoo et al. [16]), medial superior frontal gyrus (Yoo et al. [16]), parahippocampal place area (PPA) (Weiskopf et al. [15]), the anterior cingulate cortex (ACC) (Weiskopf et al. [13], Yoo et al. [16], Caria et al. [19]), insula (Veit et al. [21]), Broca's area (Rota et al. [20]), and amygdale (Posse et al. [12]). Importantly, these studies have reported evidence for behavioral modifications that accompany self-regulation training.

FMRI-BCI is a general system employing real-time fMRI technology that enables various applications including training to self-regulate activity in precisely specified regions of the brain to study plasticity and functional reorganization, application of the knowledge so derived in psychophysiological treatement, quality assurance of neuroimaging data, presurgical patient assessment and teaching of brain imaging methods (Weiskopf et al. [7]). In the context of a self-regulation experiment, fMRI-BCI can extract BOLD activity from voxels in one or more regions of interest (ROIs) in the brain to compute average activity in the ROIs, or correlation coefficient of activity between ROIs, or any other function that could be used to provide feedback to the participant. However, fMRI-BCI need not necessarily function based on self-regulation of brain activity alone. There has recently been much progress in the detection and discrimination of mental states using fMRI data (Haynes and Rees [23]). Although much of the research work has focussed on offline pattern classification of brain states using machine learning techniques, there are also attempts to develop online classification (Laconte et al. [24]). With this approach the participant does not have to be trained to regulate activity in the brain. On the contrary, the system learns to recognize the patterns of activity that spontaneously occur in a participant's brain. This new approach promises applications such as lie detection, and detection of cognitive, perceptual, and emotional states for neuroscientific research and clinical treatment. The output from such a system could also be used for communication and the control of external devices.

Section 2 presents the general architecture of an fMRI-BCI system and its components. Section 3 paints a picture of potential applications of this emerging approach for neuroscientific research. Section 4 describes possible applications in psychophysiological treatment. Section 5 offers concluding remarks.

## 2. ARCHITECTURE OF fMRI-BCI

**Figure 1:**
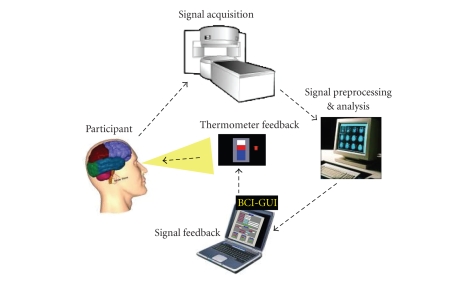
An fMRI-BCI system is a closed-loop system that has the following major components: (1) signal acquisition, (2) signal analysis, (3) signal feedback, and (4) the participant. The first 3 components are usually executed on separate computers for optimizing the system performance, and are connected together by a local area network (LAN). Spatially localized brain activity is measured by fMRI using the BOLD effect which is the neurovascular response to electric brain activity. Usually, echo planar imaging (EPI) sequences are applied to acquire functional images when the subject is performing a mental task or imagery. Images are reconstructed, distortion corrected, and averaged by the signal acquisition component. The signal analysis component retrieves the data, and performs data preprocessing, such as including 3D motion correction, and statistical analysis. The signal time series of interactively selectable regions of interest are then exported to the custom-made visualization software (signal feedback component) which provides feedback to the subject using video projection.

An fMRI-BCI system is a closed-loop system that can be depicted as shown in Figure 1. It has the following major components: (1) the participant, (2) signal acquisition, (3) signal analysis, (4) signal feedback. The last 3 components are usually executed on separate computers for optimizing the system performance, and are connected by a local area network (LAN).

Localized brain activity is measured by fMRI using the BOLD effect which is the vascular response to neural activity. FMRI signals are usually acquired by echo planar imaging (EPI). Our experiments are conducted using a 3T whole body scanner (Trio, Siemens, Erlangen, Germany) with standard head coil. EPI sequence parameters used are repetition time TR = 1.5 seconds, echo time TE = 45 milliseconds, flip angle = 70°, 16 slices, bandwidth 1.3 KHz/pixel, FOV_PE_ = 210, FOV_RO_ = 210, image matrix = 64 × 64, voxel size 3 × 3 × 5 mm^3^. Images are reconstructed, distortion corrected, and averaged on the magnetic resonance scanner computer. The signal analysis component is implemented in our work using turbo-brain voyager (Brain Innovations, Maastricht, The Netherlands) (Goebel [25]). The signal analysis component retrieves reconstructed images, and performs data preprocessing (including 3D motion correction) and statistical analysis. The time series of selected regions of interest are then exported to the custom-made visualization software which provides feedback to the subject using either a video projection or MRI compliant goggles.

Feedback is presented with a delay that depends on the time involved for image acquisition and processing. A short delay is critical (the best achieved so far is about 1.3 seconds in our lab) for volitional control. The advantage of fMRI in comparison to EEG is its superior spatial specificity and resolution. Most studies so far have used the BOLD signal from static regions of interest (ROIs) from one or multiple EPI slices of the human brain for feedback. ROI is chosen by drawing a rectangular area on the functional map computed in the signal analysis software (e.g., TBV). To improve selection of ROIs, functional maps could be coregistered with previously acquired anatomical scans of the subject. Studies have also used differential feedback (Weiskopf et al. [13], Weiskopf et al. [15]) between two ROIs to subtract out global signal changes. Specificity of the signal can be further improved by designing a protocol that includes bidirectional control, that is, both increase and decrease of the BOLD activity in the ROI. General effects of arousal and attention caused by the demands of the task or the state of the subject are thus canceled out leaving only the effects of increase or decrease of the signal.

**Figure 2:**
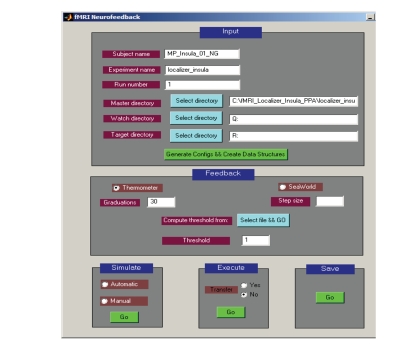
“BCI-GUI” is a software tool with a graphical user interface to configure the fMRI-BCI experiment, enter user input and protocol parameters, choose one among a variety of feedback modalities, present feedback to the subject in real-time, and report experimental results as graphs and charts at the end of the feedback session. The software is extensible, allowing development of additional preprocessing, analysis, and feedback methods. Modifications to the system can be tested offline by simulating fMRI data before bringing it to the MRI scanner.

Average BOLD values from ROIs are computed by the signal analysis software and stored in a continuously updated file to be retrieved in real-time by the signal feedback component. In our work, we have developed a custom software called “BCI-GUI” that provides a graphical user interface to configure the fMRI-BCI experiment, enter user input, choose one among a variety of feedback modalities, present feedback to the subject in real-time, and report experimental results as graphs and charts at the end of the feedback session (see Figure 2).

**Figure 3:**
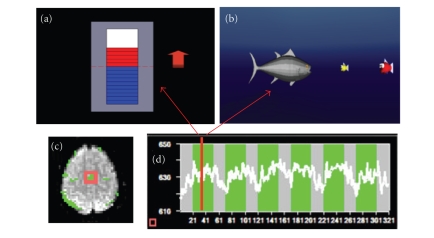
An important criterion in successfully training subjects to self-regulate their BOLD response is the feedback. (a) shows the thermometer feedback that gives regularly updated snap shot of brain activity as graduations in the thermometer. Positive BOLD activity with respect to baseline activity can be shown in one color (red) to differentiate negative BOLD activity (blue). Using this feedback the subject has an intuitive grasp of increasing or decreasing the thermometer graduations during self-regulation. (b) shows an exemplar virtual reality environment for feedback. A well-designed virtual reality feedback system can enhance the efficacy of training subjects to self-regulate a localized brain region. Volunteers have to control a 3D animated character, a fish in water, by self-regulating their BOLD response to carry out a task of moving the fish towards a food item (a smaller fish) and eating it. (c) shows the localization of supplementary motor area (SMA) as the region of interest (ROI). (d) shows a participant's time series of self-regulation of BOLD response from SMA after 3 training sessions.

Many feedback modalities, such as verbal, visual, auditory, olfactory, tactile, and a combination of these, are possible. However, most studies have used visual feedback. A variety of visualization methods have been employed by different researchers to indicate the required level of activation over time. Scrolling time series graphs and curves of BOLD activation of the ROI is a computationally fast yet effective method to provide immediate information to the subject (Weiskopf et al. [13], DeCharms et al. [14], Weiskopf et al. [15]). Sitaram et al. (Sitaram et al. [18]) introduced the thermometer type of feedback that shows a snap-shot of brain activity as variations of the thermometer. Positive BOLD activity with respect to baseline activity can be shown in one color (red) to differentiate negative BOLD activity (blue). Sitaram et al. also introduced virtual reality (VR) for feedback (Sitaram et al. [18]) (Figure 3).

## 3. fMRI-BCI APPLICATION TO NEUROSCIENTIFIC RESEARCH

### 3.1. Background

There are two general approaches in neuroscience for studying the interaction between brain and behavior. The first category involves the manipulation of the neural substrate and the observation of behavior as a dependent variable (Moonen and Bandettini [26], Feinberg and Farah [27]). The effects of stimulation and lesions of brain areas are studied with this approach. The second approach is less intrusive in nature, manipulating behavior as an independent variable and neural function as a dependent variable, constituting the psychophysiological approach.

fMRI-BCI is in a unique position to combine both approaches. It is a manipulative approach, as the subject is trained to voluntarily change the activity in a particular region of the brain as an independent variable to observe the changes in behavior. It realizes also the psychophysiological perspective as it incorporates experimental paradigms with neural response as the dependent variable. Using the EEG neurofeedback and BCI approaches, studies on slow cortical potentials (SCPs) reported behavioral effects on lexical processing, motor action, and musical performance (Rockstroh et al. [28], Pulvermuller et al. [29], Egner and Gruzelier [30]). FMRI-BCI has the advantage of targeting a localized brain region, with high spatial resolution and a reasonable temporal resolution. BOLD feedback with a latency of less than 1.3-second interval has been achieved (Weiskopf et al. [13]). We discuss below fMRI-BCI applications in emotional processing, language processing, pain processing and pain perception, motor control, sensory perception, and aversive conditioning.

### 3.2. Emotional processing

Weiskopf et al. (Weiskopf et al. [13]) used fMRI-BCI to study the effect of volitional control of anterior cingulated cortex (ACC) on emotional processing. From previous anatomical and functional studies two major subdivisions of the ACC are distinguished, which subserve two distinct types of functions. The dorsal ACC is called the “cognitive division” (ACcd) and the rostral-ventral “affective” division (ACad). Due to its involvement in different functional networks, physiological self-regulation was applied to study cognitive and emotional parameters, for example, emotional valence and arousal, dependent on the differential activation of the two subdivisions. In this study, two continuously updated curves were presented to the subject depicting BOLD activity in ACcd and ACad. During blocks of 60-second duration, subjects were instructed to move both curves upwards (alternating 60 seconds rest and 60 seconds up-regulation). The subject was instructed to use his own strategy for voluntary BOLD regulation. The subject reported that he used the imagery of winter landscapes, engaging in snowboarding and social interactions during up-regulation, and attending to the feedback curve without performing any specific imagery during the rest blocks. An improved control of the rostral-ventral affective subdivision was observed during training. Subsequent testing of the affective state using self-assessment Manikin (SAM) (Bradley and Lang [31]) showed an increase in valence and arousal during the up-regulation of BOLD in the ACad only.

**Figure 4:**
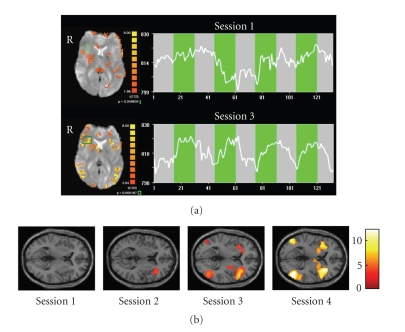
(a) Single subject statistical maps (left) and BOLD time-courses (right) of the right anterior insula in the first (upper) and in the last sessions (lower). The selected region of interest is delineated by the green box. The time course of the BOLD activity (white line) is related to the ROI selected and is showing the progress during the regulation blocks (green) and the baseline blocks (grey). Number of volumes is along the *x*-axis and magnitude of the signal is along the *y*-axis. (b) Random effects analysis on the experimental group confirmed an increased BOLD magnitude in the right anterior insular cortex over time course. SPM2 analysis of the single sessions showed no significant activation during the first session in the target area; a significant activation cluster (*t* = 4.50; *P* = .001 uncorrected) during the second session (MNI coordinates: 39,33,0); and a highly significant activation cluster (*t* = 10.23; *P* *<* .001 uncorrected) during the third session located (MNI coordinates: 36,26,6) (Caria et al. [32]).

In a recent study (Caria et al. [32], Figure 4), we investigated whether healthy subjects could voluntarily gain control over right anterior insular activity. Subjects were provided with continuously updated information of the target ROI's level of activation by the thermometer feedback. All participants were able to successfully regulate BOLD—magnitude in the right anterior insular cortex within three sessions of four blocks each. Training resulted in a significantly increased activation cluster in the anterior portion of the right insula across sessions. An increased activity was also found in the left anterior insula but the percent signal change was lower than in the target ROI. Two different control conditions intended to assess the effects of nonspecific feedback and mental imagery demonstrated that the training effect was not due to unspecific activations or non-feedback-guided strategies. Both control groups showed no enhanced activation across the sessions which confirmed our main hypothesis that rtfMRI feedback is area specific. The increased activity in the right anterior insula during training demonstrates that the effects observed are functionally specific and self-regulation of right anterior insula only is achievable. This is the first group study demonstrating that volitional control of an emotional area can be learned by training with an fMRI-BCI. We are presently conducting further studies to understand the behavioral effects of volitional control of insula.

### 3.3. Neuroplasticity of motor systems

**Figure 5:**
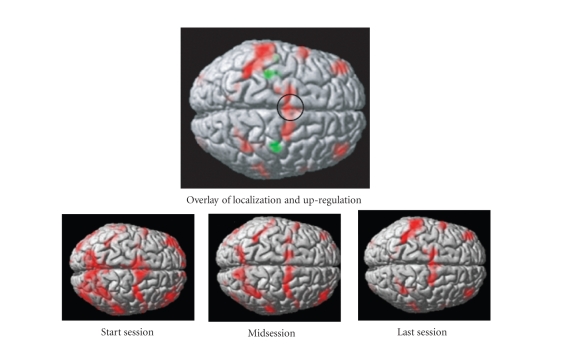
Study of neuroplasticity and functional reorganization is of much research interest. Real-time fMRI feedback could be used to successively reactivate affected regions of the brain. (a)-(d) show results of offline analysis in terms of functional activity superimposed on the anatomical structure of a healthy volunteer trained to self-regulate supplementary motor area (SMA). (a) shows significant activity around the SMA during the functional localization session when the volunteer carried out self-paced finger tapping task. (b)-(d) show brain activity during the first, middle, and last session of self-regulation training. With increased training there was a distinct reduction in activation in other areas, indicating that self-regulation training focuses on activity in the region-of-interest.

Study of neuroplasticity and functional reorganization for recovery after neurological diseases such as stroke is of relevance. Real-time fMRI feedback could be used to successively reactivate affected regions of the brain. Sitaram et al. (Sitaram et al. [18]) trained 4 healthy volunteers to control the BOLD response of the SMA. Offline analysis showed significant activation of the SMA with training. Further, with training there was a distinct reduction in activation in the surrounding areas, indicating that volitional control training focuses activity in the region-of-interest (Figure 5).

### 3.4. Language processing

Rota et al. (Rota et al. [20]) explored human capacity for differential self-regulation of the BOLD activity recorded locally in Broca's area (BA 45). The linguistic task used to localize the ROI (BA 45) was previously shown to activate the inferior frontal gyrus (Dogil et al. [33]). The task consisted of reading and manipulating the syntactic structure of German sentences. Four healthy volunteers were trained with a thermometer feedback of activity from the ROI for a total of 12 sessions in a 3T Siemens Trio with the following EPI parameters: TR 1.5 seconds, TE 45 milliseconds, flip angle = 70°, 16 slices, bandwidth 1.3 KHz/pixel, voxel size 3 × 3 × 5 mm^3^. For behavioral assessment of the effect of feedback training, two linguistic tests were performed by the volunteers immediately before and after the feedback sessions. The two tests involved grammatical judgement and emotional prosody identification. Their results showed that up-regulation of the right BA 45 correlated with emotional prosody identification.

### 3.5. Visual perception

Tong et al. (Tong et al. [34]) used fMRI to study binocular rivalry when a face and a house were presented to different eyes. As the retinal stimulation remained constant, subjects perceiving changes from house to face were accompanied by increasing activity in the fusiform face area (FFA) and decreasing activity in the parahippocampal place area (PPA), while subjects perceiving changes from face to house was seen during opposite pattern of responses. Although correlations have been found between increased brain activities in certain regions during reported conscious perception, as summarized above, a definite causal link has not been established. Is the firing activity of these neurons merely covarying with the percept? Are these cells really the central players in the percept? How tight is the link between the onset and strength of activity and the behavior on a trial-to-trial basis? We propose that fMRI-BCI can be applied to clarify these issues.

There are 3 stages to our proposed experiment: pretest, volitional control training, and posttest. In the pretest, the subject observes the rival images of houses and faces presented separately and simultaneously to the two eyes, and to press a button to indicate the changing percepts. This stage will establish the frequency and duration of the percepts. During the volitional control training, the subject's brain regions considered to be implicated in the one of the percepts (ROI) are localized. The subject is then trained in several sessions to self-regulate the ROI (i.e., FFA). During training, the subject is conditioned to decrease (or increase) the BOLD activity of the ROI of the face area. In the posttest, the binocular rivalry task is presented again to measure frequency and duration of the changing percept. If the subject has been successfully trained to self-regulate the BOLD activity in FFA, one may expect a significant change in the perception of faces. This establishes the causal link between conscious perception of the image and the brain activity in the corresponding region.

## 4. fMRI-BCI APPLICATION TO PSYCHOPHYSIOLOGICAL TREATMENT

### 4.1. Background

Behavior medicine focuses on the application of learning theories to the treatment of medical disorders. To give an example: patients with attention-deficit and hyperactivity disorder (ADHD) (Fuchs et al. [35]) were treated with self-regulation of 12–15 Hz EEG brain activity. Epilepsy patients were trained to suppress epileptic activity by self-regulation of slow cortical potentials (SCP) (Kotchoubey et al. [36]). If the neurobiological basis of the disorder is known in terms of abnormal activity in a certain region of the brain, fMRI-BCI can be targeted to those regions with greater specificity for treatment. Many types of disorders, namely, memory disorders, chronic pain, motor disorders, psychopathy, social phobia, depression, emotional disturbances, anxiety, and posttraumatic disorder might be treated with fMRI-BCI.

### 4.2. Stroke rehabilitation

A potential clinical application of fMRI-BCI is the rehabilitation of the victims of motor disorders. Hemiparesis (paralysis or weakness affecting one side of the body) is a common neurological deficit after stroke (Kato et al. [37]). Recent studies have suggested that the recovery after stroke is facilitated by the reorganization of cortical motor areas in both damaged and nondamaged hemispheres. Despite the potential of recovery, relearning of the movement of the disabled arm does not occur spontaneously. A treatment modality consists of successive reinforcement of the elements of the required behavior to activate the neural network involved in arm movement (Dobkin [38]). This might be achieved by training patients first to learn to reactivate the premotor area, and then in a stepwise fashion the primary motor cortex, basal ganglia, and cerebellum. The reorganization of the brain regions could be assisted with fMRI-BCI. Yoo and Jolesz reported successful modification of motor function in response to real-time fMRI feedback (Yoo and Jolesz [11]). Scharnowski et al. (Scharnowski et al. [39]) trained volunteers to differentially self-regulate SMA and PPA in four sessions, and then tested for effects on reaction times in a bimanual motor task. An increase of activity in the SMA only correlated with a speeded response.

We are exploring a new approach to assist movement restoration in stroke victims using fMRI-BCI. There is evidence for motor recovery and cortical reorganization after stroke when patients undergo treatment involving mental practice and mental imagery (de Vries and Mulder [40]). These results indicate that enhancing neural activity in the motor cortex is bilateral to the lesion (target ROI) while simultaneously inhibiting activity in the motor cortex contralateral (secondary ROI) to the lesion may help in stroke rehabilitation. This means that the fMRI-BCI training should aim to increase activity in the target ROI while maintaining a negative correlation with the activity in the secondary ROI. In order to achieve this, we compute online a feedback value proportional to the product of the negative correlation coefficient of the activation time courses for the specified number of time points (e.g., last 10 time points) of the two ROIs, and the magnitude of the activation in the target ROI. The feedback value computed is presented to the subject as thermometer bars or a dial indicating positive to negative correlation. Subjects can be trained to increase the bars of the thermometer to enhance activation in the ipsilesion area while inhibiting the contralesion area. Our preliminary results with 2 healthy volunteers who were provided feedback of motor imagery have shown that subjects could be trained to enhance activity in the target ROI in counter-correlation with the activity in the contralesion hemisphere. Our future work is aimed at establishing the behavioral effect of this enhanced activity and the suitability of this method for stroke therapy.

### 4.3. Treating chronic pain

Chronic pain is one of the most frequent clinical problems. Chronic pain can be substantially affected by cognitive and emotional processes (DeCharms et al. [17]). Subregions within rostral ACCr in association with other brain regions are implicated to be involved in the perception of pain. Hence, it is possible that by altering the activity in the rACC, pain perception might be accordingly varied. Indeed, Maeda et al. (Maeda et al. [41]) reported a substantial decrease of symptoms in chronic pain patients by training patients to self-regulate ACC. A further report from the same group (DeCharms et al. [17]), involving 16 healthy volunteers and 12 chronic pain patients, indicates the potential application of real-time fMRI for treating chronic pain. Subjects were able to learn to control activation in the rostral anterior cingulate cortex (rACC), a region involved in pain perception and regulation. The authors reported that if subjects deliberately induced increases or decreases of rACC fMRI activation, there was a corresponding change in the perception of pain caused by an applied noxious thermal stimulus. Control experiments showed that this effect was not observed after training without real-time fMRI feedback, or using feedback from a different region, or sham feedback derived from a different subject. Chronic pain patients were also trained to control activation in rACC and reported decreases in the ongoing level of chronic pain after training.

### 4.4. Treating emotional disorders

Emotional regulation training for patients suffering from depression, anxiety, posttraumatic disorder, and other emotional disturbances might be another application of fMRI-BCI. Experiments by Caria et al. (Phan et al. [42]) have shown that the emotional system can also be self-regulated. In another study (Posse et al. [12]) subjects were trained to self-regulate their amygdala activation by a strategy of self-induced sadness. Behavioral tests showed that the subjects' emotional ratings correlated with their activity in amygdala, substantiating the earlier findings that amygdala is involved in negative emotions (Anders et al. [43]). Weiskopf et al., (Weiskopf et al. [13]), Caria et al., (Caria et al. [19]), Veit et al., (Veit et al. [21]) have reported that volitional control of ACC and insula correlated with changes in emotional valence and arousal.

### 4.5. Psychopathy and social phobia

Criminal psychopathy is a major problem encountered by society. Psychopaths form only 15–30% of prison population, but they commit 50% more crime than nonpsychopaths (Viding [44], Viding [45]). The brain regions and neural mechanisms of the disorder are not well understood. A psychopath is characterized by poverty of affect and lack of shame, superficially charming, manipulative, and shows irresponsible behavior. Previous studies have implicated orbitofrontal cortex, amygdala, anterior insula, anterior parietal cortex and anterior cingulated cortex (Brennan and Raine [46], Blair [47], LeDoux [48]). Our studies (Veit et al. [21], Birbaumer et al. [49]) have shown that a hypoactive frontolimbic circuit may represent the neural correlate of psychopathic behavior, whereas an overactive frontolimbic system may underlie social fear. Increased activation in the emotionally relevant areas such as amygdala, anterior insula, and medial frontal cortex may lead to improved aversive conditioning. A real-time fMRI system for the specific treatment of criminal psychopathy is currently under development. Criminal psychopaths are trained to self-regulate their BOLD activity in localized brain areas implicated in the disorder, such as, anterior insula and amygdala. Behavioral effects of this training are investigated by conducting aversive delay conditioning and other behavioral tests before and after treatment.

## 5. DISCUSSION

Brain-computer interfaces based on fMRI enable real-time conditioning of circumscribed brain regions to learn volitional control of those regions. This is an emerging field of intense research excitement. Technological advancement in higher field MRI scanners, data acquisition sequences and image reconstruction techniques, preprocessing algorithms to correct for artefacts, more intelligent and robust analysis and interpretation methods, and faster feedback and visualization technology are anticipated to make fMRI-BCI widely available and applicable. Examples of such future developments are z-shimming sequence adapted for fMRI-BCI to correct for magnetic inhomogeneity differences; connectivity analysis, for example, using dynamic causal modelling (Friston et al. [50]) incorporating a whole network of neural activity instead of just one local ROI; support vector and other machine learning and pattern classification approaches (LaConte et al. [51], Mouräo-Miranda et al. [52]); independent component analysis (Esposito et al. [53]) for extracting BOLD response of interest; motion compensation (Thesen et al. [54]) for head motion artefact removal; and augmented virtual worlds for more immersive feedback. Anticipated developments in dedicated purpose MRI scanners (such as those of ONI Medical Systems, Inc, Wilmington, Mass, USA) that offer high-field performance at a low-price compared to whole body scanners can make fMRI-BCI applications more user-friendly, affordable and hence widely accessible.

There are certain limitations to the current fMRI-BCIs that future research would have to overcome. Conventional neuroimaging methods seek to find out how a particular perceptual or cognitive state is encoded in brain activity by measuring brain activity from many thousands of locations repeatedly, but then analyzing each location separately (univariate analysis). If the responses at any brain location differ between two states, then it is possible to use measurements of the activity at that location to determine or decode the state. However, it is often difficult to find individual locations where the difference between conditions is large enough to allow for efficient decoding. In contrast to the conventional analysis, recent work shows that neuroimaging may be improved by taking into account the spatial pattern of brain activity (Haynes and Rees [23]). Pattern-based methods use considerably more information for detecting the current state from measurements of brain activity. LaConte et al. (Laconte et al. [24]) have reported probably the first implementation of real-time multivariate classification that could be applied to fMRI-BCI. With such improvements, FMRI-BCI has the potential of establishing itself as a tool for certain types of neuroscientific research and experimentation, and also as an aid for psychophysiological treatment.
